# Xeroderma Pigmentosum: Ocular Findings in an Isolated Brazilian Group with an Identified Genetic Cluster

**DOI:** 10.1155/2019/4818162

**Published:** 2019-10-31

**Authors:** Maria Claudia Schelini, Luis Fernando O. B. Chaves, Marcia C. Toledo, Francisco W. Rodrigues, Tauan de Oliveira, David L. C. Isaac, Marcos Avila

**Affiliations:** Department of Ophthalmology, Federal University of Goias, Goiania, Brazil

## Abstract

**Purpose:**

Xeroderma pigmentosum (XP) is a rare autosomal recessive genetic disorder characterized by increased susceptibility to UV radiation- (UVR-) induced skin pigmentation, skin cancers, ocular surface disease, and, in some patients, sunburn and neurological degeneration. Eight different genes are affected, and the prevalence of the disease differs across the world. The present study describes the main ophthalmologic features and symptoms in patients with XP in this case series.

**Methods:**

Patients were examined consecutively at the University Hospital of the Federal University of Goias between January 2016 and June 2018. All patients underwent ophthalmologic examination and were asked about their ophthalmological history and the presence of ocular symptoms.

**Results:**

Twenty-one patients with genetic confirmation were evaluated. The genetic variants XPV and XPC were detected in the patients. The most prevalent findings include eyelid changes, observed in 80.9% of the patients, and ocular surface changes as punctate keratopathy, occurring in 16 patients (76.2%), corneal neovascularization, and corneal opacities. Six patients (28.5%) presented corneoconjunctival tumor. More than half of patients had previous history of treatment of ocular neoplasia. Ocular burning was the most reported symptom.

**Conclusions:**

The ocular characteristics identified in this study corroborate the existing literature, mainly related to the surface. Concerning the XP variant and the gravity of ocular signs, XPC has earlier and more severe symptoms than XPV. Due to their relative rarity, publications of XP cases are important to understand the possible damages caused by the disease in the eyes and surrounding area.

## 1. Introduction

Xeroderma pigmentosum (XP) is a rare autosomal recessive genetic disorder first described by Hebra and Kaposi in 1874 [1]. It presents variable prevalence, with estimates of 1/1,000,000 in North America [2], 1/22,000 in Japan [3, 4], and 1/10,000 in Tunisia [5].

The disease causes the patient to be susceptible to UV damage. It occurs due to a protein-enzymatic deficiency responsible for the repair of damaged DNA induced primarily by ultraviolet (UV) radiation [6, 7]. This primary defect leads to an increased susceptibility to UV radiation- (UVR-) induced skin pigmentation, skin cancers, ocular surface disease, and, in some patients, sunburn and neurological degeneration [8], with the development of cutaneous and systemic neoplasms [9, 10]. Eight different genes are affected, which were classified as complementary groups (XP-A, B, C, D, E, F, G, and XPV) [10, 11]. The prevalence of these groups differs across the world, and groups A, C, D, and V are most common in the United States and Europe [7, 10]. Complementary groups from A to G show deficiency in the repair of the DNA formation/transcription phase, and the complementary group XPV (or variant) occurs because of a defect in the postreplication repair, due to the deficiency of the enzyme polymerase POLH [6]. XP patients in which TC-NER remains intact (groups C, E, and V) do not, in general, suffer from neurological problems and have normal sunburn reactions, whereas those in groups in which both sub-branches of NER are affected often develop neurological problems and have abnormal sunburn reactions [8].

Brazil lacks population-based studies that assess the epidemiological situation and the real impact of XP. Chaibub et al. [12] reported XP in an isolated area in central-western Brazil (village of Araras, municipality of Faina, and state of Goias), where 21 patients were diagnosed with XP, indicating the possibility of a genetic cluster [13].

Previous studies have described ocular changes in at least 40% to 100% of patients affected by XP. Blepharospasm and photophobia were the most common symptoms [7, 10, 14]. The most commonly affected ocular structures are the eyelids, conjunctiva, cornea, and sclera, i.e., areas most exposed to sunlight [14, 15].

The present study describes the main ophthalmologic features and the presence of suspected malignant lesions in the cluster of patients with genetically typed XP. This is the first cross-sectional descriptive study of a single Brazilian population group affected by the disease.

## 2. Materials and Methods

This is an observational, cross-sectional case series describing the findings of ocular involvement in patients with XP from a single genetic cluster living in a community in the state of Goias, Brazil.

Patients were evaluated consecutively at the University Hospital of Federal University of Goias, Brazil, between January 2016 and June 2018. They presented with XP that was genetically confirmed by the group of Munford et al. [13] and will not be repeated here.

The study was approved by the Research Ethics Board of the Federal University of Goias (UFG) Hospital das Clinicas, and informed consent was obtained from the patients or caregivers. The study adhered to the principles of the Declaration of Helsinki.

All patients underwent ophthalmological examination and were asked about their ophthalmological history and about the presence of ocular symptoms, such as photophobia, dry eye/foreign body sensation, and ocular burning. The ophthalmological examination included the measurement of the best corrected visual acuity (BCVA), measured with Snellen chart, biomicroscopy, and fundoscopy, by indirect binocular ophthalmoscopy. Additional testing included tear film break-up time (BUT) and the Schirmer I test. Fluorescein dyes (Fluorescein, Allergan Produtos Farmacêuticos LTDA, São Paulo, Brazil), rose bengal 1%, and toluidine blue 1% (Ophtalmos®, São Paulo, Brazil) were utilized. The Schirmer test was performed without anesthetic eye drops (Schirmer I-Ophtalmos®, São Paulo, Brazil). Values below 5 mm were considered an aqueous deficiency in lacrimal production, and values between 6 mm and 10 mm were considered as borderline deficits [16]. BUT evaluation was performed with 1% fluorescein. Values that were less than 10 seconds were considered abnormal. The result after the instillation of rose bengal dye was rated positive from any number of stained areas or spots. Toluidine blue was instilled in patients who had detected surface alterations as pinguecula, pterygium, or corneoconjunctival tumors, and was defined “positive” as the presence of homogeneous staining without stippled appearance, and diffuse distribution of dye uptake was evident.

The obtained data were processed with SPSS software for Windows version 21.0 (Statistical Package for the Social Sciences, SPSS Inc., Chicago, IL, USA). To verify the normality of the variables, the Kolmogorov–Smirnov test and the Shapiro–Wilk test were applied. In both tests, variables with values of *p* > 0.05 were considered within a normal distribution. Qualitative variables were presented in absolute and relative values, with differences analyzed by Fisher–Freeman–Halton test with bilateral probability estimated by the Monte–Carlo method. The quantitative variables are mean, standard deviation, minimum, median, and maximum.

## 3. Results

Twenty-one patients with genetic confirmation were evaluated. Of these, 8 (38%) were female. The mean age was 42.8 years (SD = ±20.4), with a median of 40 years (range = 14–83). The genetic variant XPV was detected in 18 patients (85.7%), 14 of them homozygous, while in the other 3 patients, XPC (14.3%) was detected. The mean minimum age for onset of ocular symptoms was 20.1 ± 16.3 years, with a median of 15 (6–60) years. Five patients (23.8%) reported no ocular symptoms and 4 reported occasional symptoms (19.0%).

Eighteen (86%) of the 21 patients in the study had a previous history and skin tumor excision (SCC, BCC, melanoma), 15 (71%) reported eyelid tumors, and 8 (38%) had a history of ocular surface lesion. However, it was not possible to define the nature of the lesions as many had already been removed surgically at different hospital services with no biopsy results. Regarding the ocular surface lesions, it is also not possible to state whether they were lesions such as pterygium or some premalignant or malignant lesion.

**Figure 1:**
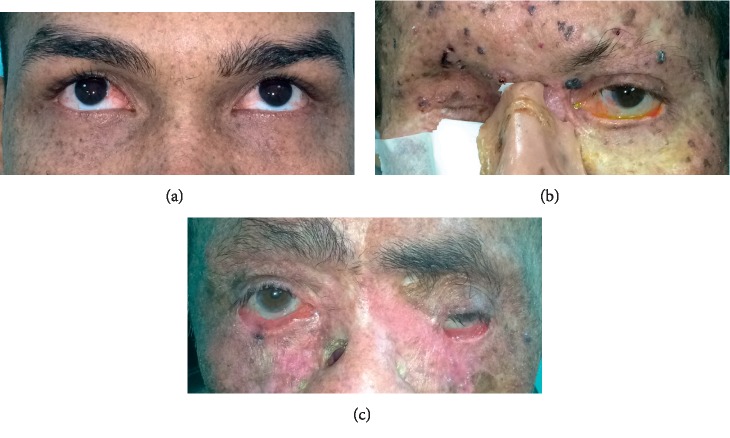
Patients with xeroderma pigmentosum (XP)-V. (a) A 25-year-old patient with mild ophthalmic presentation, ocular hyperemia and pterygium. (b) A 48-year-old patient presents with anophthalmic cavity secondary to exenteration due to malignant tumor, and to the left, eyelid irregularity, ectropion, lagophthalmos, and ocular hyperemia. (c) A 81-year-old patient with lower eyelid retraction bilaterally and history of bilateral inferior eyelid tumor excision and had a nasal prosthesis with a superior orifice.

**Figure 2:**
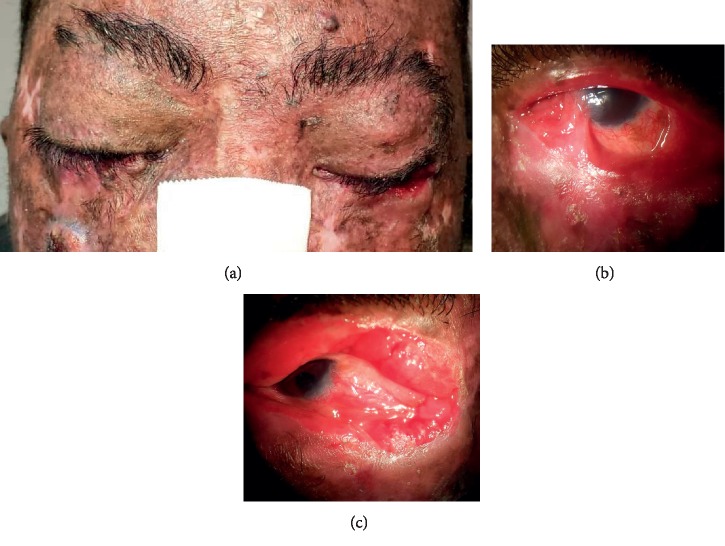
Patient XPC, 24 years old, with ocular surface and eyelids involvement.

Eyelid changes were observed in 17 patients (80.9%). The findings were irregularities in eyelid border in 11 patients (52.3%), actinic keratosis in 10 patients (47.6%), ectropion in 7 patients (33.3%), lagophthalmos in 7 patients (33.3%), telangiectasia in the eyelid border in 5 patients (23.8%), madarosis in 5 patients (23.8%), lacrimal point stenosis in 5 patients (23.8%), trichiasis in 2 patients (9.5%), and entropion in 1 patient (4.7%). By the time of the examination, 4 (19%) patients had eyelid lesions suspected of basal cell carcinoma or squamous cell carcinoma. The most common change detected on the slit-lamp examination was blepharitis (hyperemia and crusting of the eyelids) (90.5%). Some characteristics are shown in Figures 1 and 2.

**Figure 3:**
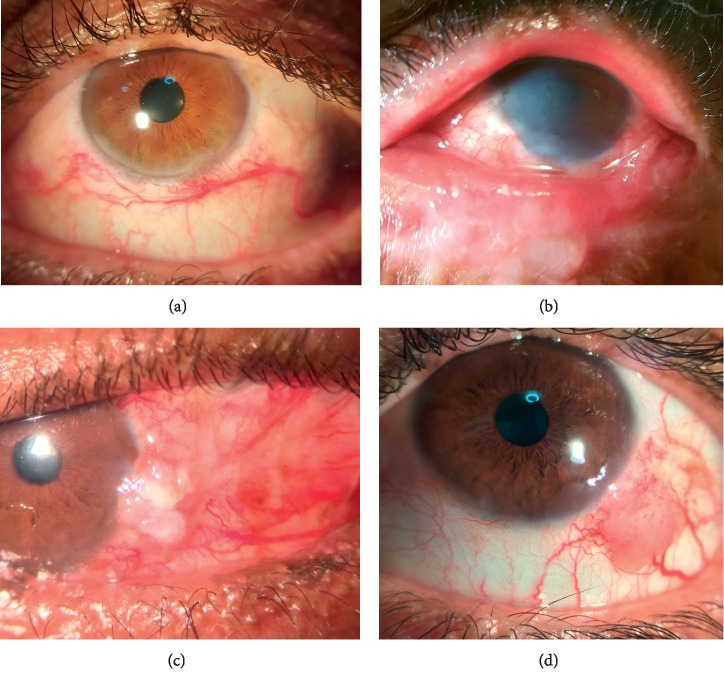
Changes in the ocular surface. XPV patients, presenting vascular tortuosity (a) and corneal conjunctival tumors (c, d). XPC patient (b), with corneal opacification and eyelid changes.

The most frequent ocular surface finding was punctate keratopathy, occurring in 16 patients (76.2%). Six patients (28.6%) had corneal neovascularization, and 3 patients (14.3%) had corneal opacities (Figure 3). Six patients (28.5%) presented corneoconjunctival tumor, and toluidine blue test was positive in 4 of these patients. Ten (47.6%) had pterygium, 9 (42.8%) had interpalpebral melanosis, and 6 (29%) had pinguecula. Seventeen patients (80.9%) presented BUT within 10 seconds. The Schirmer test was altered in 5 patients (23.8%). Rose bengal staining was positive in 12 (57.1%) of the 21 patients. The main ocular findings and symptoms according to the genetic group are described in Table 1.

Four patients (19%) had a diagnosis of glaucoma and two patients (9.5%) had chorioretinal scars, suggestive of previous ocular toxoplasmosis. Eight patients (38.1%) had cataract or had undergone cataract surgery. Two patients (9.5%) had anophthalmic cavity (exenteration by SCC), and 1 patient (4.7%) had phthisis bulbi (postcataract surgery). Five patients (23.8%) had low visual acuity, two of them due to cataracts and the other three resulting from corneal changes; one patient had macular chorioretinal scarring suggested of toxoplasmosis infection. Only one (4.7%) of the 21 patients presented bilateral blindness criteria, due to cataracts.

## 4. Discussion

The described cluster was initially discovered in 2011 by the dermatologist Chaibub in an isolated village, Araras, in the state of Goias, Brazil [12]. This community was isolated due to a disease that most of its members suffered that was believed to be contagious. Consequently, two families with a similar condition, coming from different places within that state, grouped together.

Munford et al. [13] detected in the study group 2 new express mutations of different origins, one at the donor site of intron 6, and the other at exon 8, that is, two different alleles of mutated POLH genes were recognized. These mutations may be sporadic or a consequence of the genetic crossover. Genetic changes in XP are complex, and their study can individualize patient follow-up. Eighteen subjects were identified as patients with XPV. Worldwide, the variant form of XP is responsible for approximately 10 to 20% of all XP patients [7]. In Japan, XP complementation group A (XP-A) is the most common, followed by XPV, whereas XPC is the most common in the United States and Western Europe [4]. In the present study, a prevalence of 85.7% of patients with this genetic configuration was observed. Village residents say that a large number of individuals (about 30) died with a similar pathological condition, but there is no confirmed diagnosis. This higher prevalence can be explained by the group's isolation, which, with successive generations of consanguineous marriages.

It should be noted that 17 patients were born in the village, while 3 XPC patients and 1 XPV patient came from other cities when they were older. As mentioned, phenotypic differences have been reported between the 8 XP complementation groups. XP patients from complementation groups C, E, and V (where TC-NER is preserved) have normal sunburn reactions for skin type and do not develop manifest neurodegeneration [8, 14]. XP patients from complementation groups A, B, D, F, and G (where TCNER is impaired) have severe and exaggerated sunburn reactions on minimal sun exposure and suffer neurodegeneration [8]. This group decided to analyze all the patients and describe it in one single paper.

The mean age of onset of ocular symptoms in this study was 20 years (range of 6–60 years), corroborating Brooks [7](17 years) and Alfawaz and Al-Hussein [20] (19 years) and compared with the study by Kraemer et al. [10] (4 years). This can be attributed to the fact that, although symptomatic, the search for a tertiary service happens after more evident manifestations of a disease, that is, when they are visible to the naked eye. This hypothesis is also suggested by Fassihi and Sethi [8], who correlated the severity of the disease and its diagnosis. When cutaneous disease presents itself in childhood, the earlier the diagnosis and the overall patient care take place the better, as occurs in individuals with genotypes XP-A, B, D, F, and G. However, in individuals with genotypes XPV, C, and E, who show milder effects to UV lesions, the disease may be underdiagnosed, and as a consequence, ophthalmological care is also provided late.

In the study by Fassihi and Sethi, there is a group of 12 XPV patients and 28 XPC patients. The data resemble the present article. The XPV group here presented some differences within, but, overall, they had mild skin and ocular manifestations. However, as they manage to reach a later age, new tumors can appear, and sun exposure is not always prevented, so aggressive tumors may emerge over time. Since these individuals have normal sunburn reactions for skin type and have no neurological abnormalities, typically they are not diagnosed until their second or third decade at the earliest. At this point, they have accumulated years of UVR-induced mutations, leading to development of multiple skin malignancies at a later age. XPV patients develop hundreds of skin cancers, but ocular disease is relatively mild compared with that found in XPC patients [8].

The 3 XPC patients in our study were diagnosed in childhood, and two of them were siblings. They had more skin abnormalities and history of skin tumors than the XPV. These 3 patients were 14, 23, and 26 years old, while in the XPV group, some of them ranged from 15 to 83 years. Skin cancers are seen relatively early in XPC patients. The youngest patient, a 14-year-old girl, had diagnosed multiple skin melanomas and had lack of ocular findings or symptoms. Although there were no cases of neurologic or psychiatric abnormalities, this young patient had a dull behavior. The two XPC siblings showed the first cutaneous signs—freckle-like hyperpigmentation—around 6 years of age—and ocular changes occurred around 15 years of age, but progressively more severe when compared to XPV patients. A remarkable and previously unreported observation that progressive eye disease in patients with XPC has shown to be much more severe than in other groups, with eye disease scores increasing with advancing age [8].

In the study by Brooks et al., at least 1 ocular abnormality was found in 91% of the patients in a group of 87 individuals with XP evaluated between 1964 and 2011. In the present study, alterations were found in 90.5%. In at least 71.4% of the patients, an ocular symptom was mentioned (ocular burning). Although very quoted, the ocular symptoms are very general and found with great frequency in a lot of general ophthalmology, and they are quite nonspecific. Burning, tearing, and photophobia were the most common complaints. Also, the study by Brooks et al., blepharitis was observed in 23% of the evaluated patients and ocular cancer in 10% of the cases [7]. In the present study, blepharitis was observed in 90.5%. A possible explanation for the findings is the fact that the patients evaluated in the present study were older (median age of 40 years varying from 14–83 years) than those assessed in the Brooks study (median age of 17 years and variation from 1.3 to 63.4 years). Studies on meibomian gland dysfunction and blepharitis show a higher prevalence of meibomian dysfunction in older patients [17].

Of the 21 patients examined, 6 (28.5%) (1 XPC and 5 XPV) had corneoconjuntival tumors at slit-lamp exam, and toluidine blue test was positive in 4 of these patients, but this had no significant association. Conjunctival tumors presented as a unilateral vascularized gelatinous limbal mass, or leukoplakia, located in the sun-exposed interpalpebral fissure medially or laterally, with other features as tortuous dilated feeder vessels, and foamy infiltration of the adjacent corneal epithelium (Figure 3) [18]. Of these 6 patients, 2 used topical chemotherapy (1XPV with mitomycin-C, 1XPV with interferon alfa 2-beta) [19] and had complete regression of the lesion with no significant side effects; 1 underwent topical treatment with interferon alfa for regression of extensive lesion of eyelid touching the cornea, then biopsy-evidenced squamous cell carcinoma (XPC), 1 with limbal intraepithelial neoplasia, and 2 with conjunctival intraepithelial neoplasia (XPV). Three months after surgical removal, 1 patient (XPV) had symblepharon despite measures of avoiding it. This occurrence suggests that there may be benefit in topical treatment of surface tumors before surgical treatment to prevent ocular surface scar formation.

Alfawaz and Al-Hussein [20], in a study performed in a tertiary hospital in Saudi Arabia, demonstrated a 44% prevalence of conjunctival tumors and 25.9% prevalence of malignant eyelid tumors. The present study showed a prevalence of ocular surface malignancies in 29% and 19% eyelid tumors of the evaluated patients. In both studies, the prevalence of malignant eyelid and conjunctival neoplasms was higher than that observed by Brooks et al. [7](10%) and in the British study by Lim et al.[14], which showed ocular involvement in at least 65% of patients and a history of neoplastic lesions of the surface and periocular region in 2% and 11%, respectively. A possible explanation for the increased frequency of ocular malignancies is that the increased incidence of ocular cancer may be related to the higher sunlight incidence in countries such as Brazil and Saudi Arabia when compared to the United States and United Kingdom.

On abnormalities of the surface and tear film, BUT change was observed in 80.9% of patients with values below 10 seconds (mean of 5 sec), whereas in the study by Brooks et al., such alteration was identified in 66% of the cases. Lacrimal production evaluated by the Schirmer I test showed abnormal results in 23.8% (5 patients). As lacrimal production is normal in most patients, altered results of the BUT, fluorescein test, and rose bengal staining, showing punctate keratopathy in 16 patients (76.2%), may suggest that surface changes could be explained by the irregularity and atrophy of the palpebral borders, especially of the lower eyelid, which increases ocular exposure and surface dryness. Besides, blepharitis present in approximately 90% of patients may aggravate tear (or tear film) stability. The rose bengal test, positive in 57.1% of patients, justifies the most frequent complaint of ocular burning, a common symptom in dry eye, in this case, evaporative. No cases of ocular perforation have been seen.

Remarkable among eyelid changes is the eyelid border irregularity presented in 11 patients, but it is an inaccurate finding because this feature may be related to surgical procedures, blepharitis, and tumors. Of these 11 patients, 4 had already had eyelid surgery, while the other 7 had blepharitis. Also, there was coexistence of ectropion and lagophthalmos in some of these patients. This set of simultaneous findings leads to ambiguous analysis, making it complex to define which disorder came first. The presence of palpebral tumors was associated with patients presenting lagophthalmos, and corneal scarring is found only between the XPC and XPV groups (*p* < 0001)—this can suggest that the chronic exposure of the inferior lid could contribute with the development of tumor lid and may be related to various eyelid changes secondary to surgery or chronic eye exposure.

Although blindness is not frequent, patients with XP have high morbidity due to periocular changes and recurrences of surface tumors. BCVA in the better eye was assessed to be at least 20/40. Five (24%) patients with recorded BCVAs worse than 20/40 in their better eye, were related to corneal opacity only (2 XPC, 1 XPV) and cataract (2 XPV). The youngest XPC patient's ability to comprehend and perform VA tests was compromised due to her lack of interaction.

Fundoscopic findings were not common here, as also reported in the current literature, and do not justify low visual acuity in the long term. Protection from UV rays by the anterior segment is frequently mentioned in several studies [1, 7, 10, 20]. The changes observed in this study, such as chorioretinal scarring indicative of previous infection by toxoplasmosis and glaucomatous optic neuropathy, seem to have no relation to the genetic defect itself, being acquired diseases. Neurological involvement is described in the XP-A group, especially in Japan [4], but in these series, no optic neuropathy or abnormal neurological manifestation were found, except for 4 patients with clinically treated increased intraocular pressure and glaucoma.

**Figure 4:**
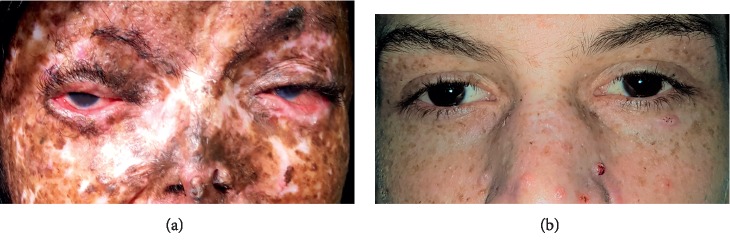
(a) Patient XPC, 26 years old. (b) Patient XPV, 20 years old.

**Figure 5:**
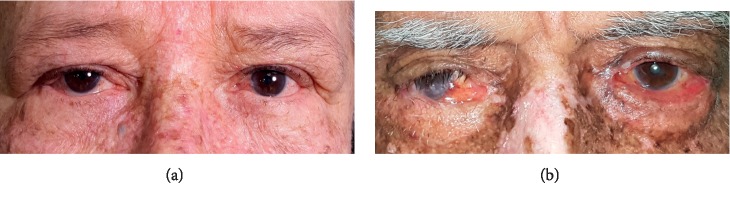
XPV patients: (a) 68-year-old woman; (b) 83-year-old man.

A feature of these patients is the heterogeneous phenotypic manifestations, varying from mild to moderate cases, despite the great exposure to sunlight throughout the patient's lifetime, common to all—rural workers since childhood. Some young patients already have an advanced cutaneous condition, yet others have reached advanced ages, above 70 years (Figures 4 and 5). This characteristic of patients with xeroderma is widely reported in studies with larger groups of patients [1, 2, 6, 7, 9, 10, 14, 15, 21]. When not identified early, clinical treatment becomes limited, leaving surgical treatment as an alternative. The ocular characteristics identified in this study corroborate the existing literature, mainly related to the surface. Although the sample size of XPC patients is very small, XPC patients had statistically more corneal involvement as well as eyelid tumors than XPV patients. They were also found to have more eyelid border irregularity (albeit not statistically significant).

Health-care planning for patients with XP requires multidisciplinary follow-up for early detection of neoplasms, genetic and family counseling, improvement in the quality of life, and emphasis on education and sun protection, which is still the best form of cancer prevention. Wearing sunglasses, hats, sunscreen, and avoiding daytime outdoor activities are the measures available so far. The future of these patients depends, in part, on their persistence in adopting preventive measures, but also on encouraging research in the quest for accurate and individualized therapies.

## 5. Conclusions

In this case series, one can observe aspects that are both similar and conflicting with the available literature. Concerning the XP variant and the gravity of ocular signs, XPC has earlier and more severe symptoms than XPV. Due to their relative rarity, publications of XP cases are important to understand the possible damages caused by the disease in the eyes and their surroundings. The report on this cluster of patients with XP in an isolated region in central Brazil may contribute to that understanding.

## Figures and Tables

**Table 1:** Ocular findings and symptoms associating with the identified mutation.

	XPV HOM (*N* = 14)	XPV HET (*N* = 4)	XPC (*N* = 3)	Total (*N* = 21)	*p*
*Description*					
Males	10 (71.4)	2 (50.0)	1 (33.3)	13	0.619

*Symptoms*					
Burning	10 (71.4)	2 (50.0)	3 (100.0)	15	0.524
Tearing	8 (57.1)	1 (25.0)	3 (100.0)	12	0.476
Photophobia	6 (42.9)	2 (50.0)	2 (66.7)	10	0.905
Itching	8 (57.1)	1 (25.0)	1 (33.3)	10	0.762
Foreign body sensation	8 (57.1)	0 (0.0)	1 (33.3)	9	0.238
Ocular pain	0 (0.0)	0 (0.0)	0 (0.0)	0	NA

*Signals*					
Corneoconjunctival tumors	4 (28.6)	1 (25.0)	1 (33.3)	6	1.000
Eyelid tumor	2 (14.3)	0 (0.0)	2 (66.7)	4	0.048

*Low visual acuity*					
Yes	3 (60,0)	0 (0,0)	2 (40,0)	5	0,190
No (better than 20/40)	11 (68,8)	4 (25,0)	1 (6,3)	16	
Blepharitis	13 (92.9)	3 (75.0)	3 (100.0)	19	0.429
Border irregularity	5 (35.7)	3 (75.0)	3 (100.0)	11	0.190
Ectropion	4 (28.6)	1 (25.0)	2 (66.7)	7	0.762
Entropion	1 (7.1)	0 (0.0)	0 (0.0)	1	1.000
Punctate keratopathy	11 (78.6)	2 (50.0)	3 (100.0)	16	0.667
Lagophthalmos	4 (28.6)	1 (25.0)	2 (66.7)	7	0.762
Trichiasis	1 (7.1)	1 (25.0)	0 (0.0)	2	0.429
Madarosis	4 (28.6)	0 (0.0)	1 (33.3)	5	0.571
Tear point change	3 (21.4)	0 (0.0)	2 (66.7)	5	0.190
Conjunctival hyperemia	10 (71.4)	4 (100.0)	2 (66.7)	16	0.714
Pinguecula	5 (35.7)	1 (25.0)	0 (0.0)	6	0.905
Pterygium	4 (28.6)	2 (50.0)	1 (33.3)	7	0.905
Symblepharon	3 (21.4)	0 (0.0)	2 (66.7)	5	0.190
Corneal opacity	1 (7.1)	0 (0.0)	2 (66.7)	3	<0.001
Corneal neovascularization	4 (28.6)	0 (0.0)	2 (66.7)	6	0.238
Cataract	4 (28.6)	0 (0.0)	0 (0.0)	6	0.238
Absence	7 (53,8)	3 (23,1)	3 (23,1)	13	0,762
Presence	4 (100,0)	0 (0,0)	0 (0,0)	4	
IOL	3 (75,0)	1 (25,0)	0 (0,0)	4	

*Tests*					
BUT	14 (100.0)	4 (100.0)	3 (100.0)	21	NA
Toluidine blue	4 (28.6)	0 (0.0)	2 (66.7)	6	0.238
Rose bengal	8 (57.1)	1 (25.0)	3 (100.0)	12	0.476
Schirmer	3 (21.4)	0 (0.0)	0 (0.0)	3	1.000

NA: not applicable; N: absolute frequency; %: relative frequency; HOM: homozygous; HET: heterozygous; XPV = variant subtype; XPC = subtype C; BUT: break-up time. All frequencies refer to the column. Statistical test used was Fisher–Freeman–Halton test.

## Data Availability

The data used to support the findings of this study are included in the article.
